# Eco-friendly magnetic activated carbon nano-hybrid for facile oil spills separation

**DOI:** 10.1038/s41598-020-67231-y

**Published:** 2020-06-24

**Authors:** Hassan Shokry, Marwa Elkady, Eslam Salama

**Affiliations:** 1 Electronic Materials Researches Department, Advanced Technology and New Materials Research Institute, City of Scientific Research and Technological Applications (SRTA-City), New Borg El-Arab, Alexandria, 21934 Egypt; 2 Environmental Engineering Department, Egypt-Japan University of Science and Technology, New Borg El-Arab City, Alexandria, Egypt; 3 Fabrication Technology Researches Department, Advanced Technology and New Materials and Research Institute, City of Scientific Research and Technological Applications (SRTA-City), Alexandria, 21934 Egypt; 4 Chemical and Petrochemical Engineering Department, Egypt-Japan University of Science and Technology, New Borg El-Arab City, Alexandria, Egypt; 5 Environment and Natural Materials Research Institute, City of Scientific Research and Technological Applications (SRTA-City), Alexandria, 21934 Egypt

**Keywords:** Pollution remediation, Nanoparticles

## Abstract

This work focuses mainly on environmental concern and protection through providing beneficial use of waste biomass from water hyacinth to produce economical nano-magnetic adsorbent material-efficient for facile oil spill separation via an external magnetic field. The water hyacinth roots showed higher oil spills adsorption affinity of 2.2 g/g compared with 1.2 g/g for shoots. Nano-activated carbon was successfully extracted from the roots of water hyacinth after alkaline activation and followed by zinc chloride treatment before its carbonization. Nano-magnetite was induced into the activated carbonized nanomaterials to synthesized nano-magnetic activated carbon hybrid material (NMAC). X-ray diffraction elucidated the crystalline nature of both extracted activated carbon from water hyacinth and its magnetic hybrid material. Scanning electron microscopic micrographs implied the nano-size of both prepared activated carbon and the magnetite hybrid materials. The magnetic properties of the fabricated nano-magnetic activated carbon were evaluated using the vibrating sample magnetometer. The magnetic nano-hybrid material recorded a maximum oil adsorption affinity of 30.2 g oil/g. The optimum oil spill of 80% was established after 60 min in the presence of 1 g/L of magnetic nano-hybrid. The magnetic nano-hybrid material that absorbs oil spills was separated from the treatment media easily using an external magnetic field.

## Introduction

Shoreline and offshore waters can be contamiend by oil drilling, accidents including oil tankers, processes, runoffs from offshore oil explorations and productions. These oil spills have a harmful impact on human health, fauna and onnatural flora. Therefore, it is an essential issue to develop new technologies for remediation the spilled oil on water^1^.

Physical, chemical and biological processes can be used to remove the oil or to destroy it *in-situ*. These methods involve oil booms, dispersants, skimmers and sorbents. Most of these techniques are costly and ineffective for sorption oil trace level^2^.

Among the different available methods utilized for oil decontamination from a water surface, adsorption is considered one of the most prominent techniques for oil spillage treatment in the presence of ambient conditions. The oil sorbent materials can be classified as inorganic mineral materials, organic natural sorbents and organic synthetic sorbents. The most important characteristics of oil sorbent materials are their hydrophobicity, the surface area and the sorbent materials capillarity. Despite the organic oil sorbent materials characterized by most of these properties, however, they are non-biodegradable and non-environmentally friend. So, they are substituted by natural sorbents such as rice straw, cotton, peat moss, cotton grass, kapok and water hyacinth have been examined as ecofriendly sorbents for spilled oil. Besides, these agricultural-based materials are inexpensive. Some of these agricultural products are waste materials, so, their reuse will save waste disposal fee^3^.

Water hyacinths (Eichhorniacrassipes) are classified as agricultural waste plant due to its rapid growth rate with huge quantities that have a negative impact on the aquatic life. However, this plant has a high absorption affinity for the decontamination of the soluble toxic matter from polluted water. So, many researchers considered it as natural biosorbent materials, especially it attains many other amazing properties such as its low cost, availability and reusability^4^.

Recently, production of highly porous activated carbon with large surface area from agricultural wastes has been focused on more environmental concerns and protection^5–7^. So, the utilization of a water hyacinth plant that considered one of the serious agricultural waste plants as a precursor for activated carbon production will support the environmental protection issue. Accordingly, the production of nano-magnetic activated carbon (NMAC) from water hyacinth has potential economic and environmental impacts. First, it converts unwanted, low-value aquatic plants to useful and high-value sorbents. Second, the production of NMAC represents novel adsorbent material for water purification. The general method of producing NMAC is based on carbonizing, activating and magnetization of the water hyacinth segments. The activation process may be achieved either physically through the carbonization process or chemically by chemical agents’ impregnation^8^.

This examination aims to achieve two positive effects with regards to environmental safety management. First, the consumption of harmful waste plants (Water hyacinths) as a precursor for production novel bio-sorbent material. This bio-sorbent material will be utilized in decontamination oil spills from polluted water and classified as environmentally friendly material. Accordingly, the utilization of water hyacinth as a precursor for production oil spill adsorbent material will participate in solving the oil pollution problems in water using an agricultural waste plant. So, this innovative work is targeted to the synthesis of green nano-magnetic activated carbon from harmful agriculture waste using a chemical facile technique that characterized by high surface area, low cost and high efficiency toward pollutant adsorption. Moreover, to facilitate the handling of this highly economical material at the wastewater remediation process, it was immobilized with nano-magnetite particles to be converted to novel magnetic material easy to be handled with external magnetic force. Accordingly, nano-activated carbon has been extracted from the water hyacinth materials through carbonization the two different water hyacinth segments (roots and shoots) either in their raw or chemically modified states to improve their oleophilic and hydrophobic characteristics. The carbonization process was accomplished by chemical activation for the different water hyacinth driven sorbent materials using either H_3_PO_4_ or ZnCL_2_ before the carbonization process. The oil sorption affinity of all produced materials was compared using the batch technique to elucidate the most efficient material. Finally, the experimental data were modeled using different kinetics and equilibrium models to describe the oil sorption process.

## Results and Discussion

### Water hyacinth as an adsorbent material

**Figure 1:**
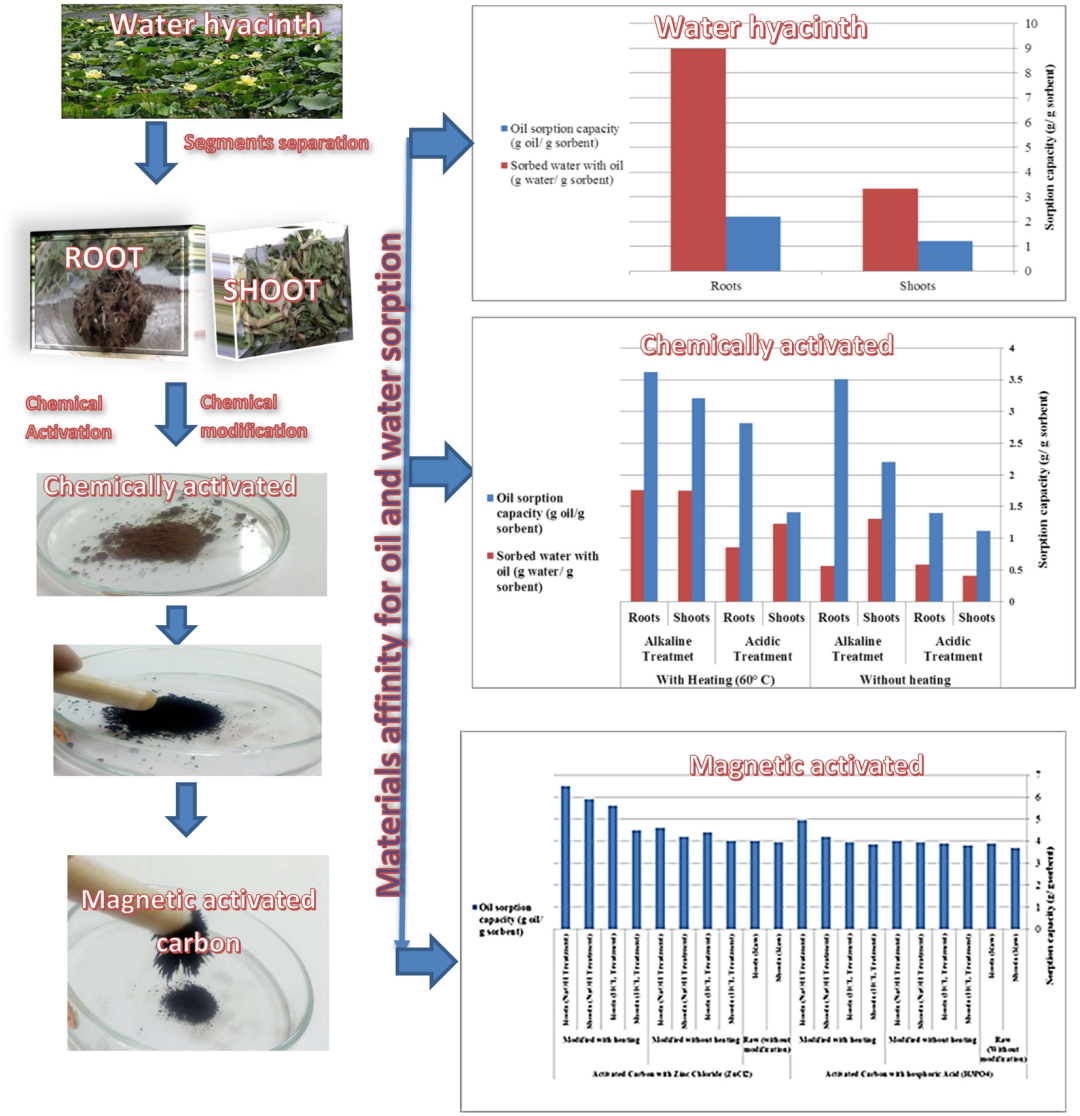
Schematic diagram for preparation of magnetic activated carbon from water hyacinth and the oil and water sorption affinity of the produced material.

The oil spill sorption was tested using the two different segments of water hyacinth (raw shoots and roots). It was indicated from Fig. 1 that the oil sorption affinity using the root segment of water hyacinth is higher than that using the shoots segment.

This is due to the dried water hyacinth roots contain much higher fiber within their internal configuration (65% fiber) than the aerial water hyacinth tissues (49% fiber). These fibers have a greater affinity for adsorption and thus, they have a high sorption affinity for oil adsorption. Besides that, water hyacinth roots characterized by their decomposition much more slowly than aerial water hyacinth tissues, providing a good mechanical strength for the sorbent material which makes it capable of being reused several times through the adsorption process. Moreover, the raw root segment of water hyacinth has a great affinity for water of about 8.98 g water/g sorbent and pick it quickly as soon as the fibers were exposed to water. This is due to the roots are considered the submerged part of the water hyacinth plant and the presence of these parts beneath the surface of the water makes their fibers have a greater tendency to adsorb water more than aerial tissues^9^.

### Effect of chemical modification process on water hyacinth sorption properties

The chemical activation of the two raw segments in the presence of either alkaline or acidic treatment induces a new exchangeable (H^+^) or (OH^-^) ions in the sorbent material according to the treatment process which improves its active surface area that by its role improve its oil sorption affinity. Figure 1 represents the sorption affinity and water pickup of the alkaline and acidic chemically modified water hyacinth, with and without heating. It was found that the alkaline chemical treatment of the powdered water hyacinth roots under heating at 60 °C gives the highest oil sorption affinity, this is maybe regarded as the chemical composition of water hyacinth fibers that consist of about 25% cellulose and 35% hemicellulose. These cellulosic fibers are insoluble in most solvents because of their crystalline nature, but they are rapidly dissolved in the NaOH solvent, where sodium hydroxide may swell cellulose and may even dissolve cellulose fiber. As the fibers swell, the yielded internal stresses will break the intermolecular bonds, leading to an increase in surface area of the fibers^10^. These results decided that the alkaline treatment at the 60 °C improves the swelling rate of fiber rapidly that facilitate the treatment process. This result was confirmed from the previous research work that stated the maximum cellulose fiber swelling is obtained within a temperature range of 50–60 °C. Below this temperature; the stiff lignin restricts the swelling that hindered the treatment process^11^.

As regards the water pickup (Fig. 1), implies that modified water hyacinth has a much lower affinity for water pickup than the raw water hyacinth. The water pickup for the modified water hyacinth ranges from 0.41 to the maximum value of 1.76 g water/g fiber, which are represented very low values compared with the water pick up in case of using the raw water hyacinth. It means that the chemical modification of water hyacinth makes it more oleophilic and less hydrophilic, and thus improves the oil sorption performance of the sorbent material.

### Effect of carbonization and magnetization processes on water hyacinth sorption properties

Generally, carbonization and magnetization processes, enhance the oil sorption affinity of the produced material compared with its parent material. This is due to the chemical activation that increases the micropores and macropores of the produced activated carbon, where most of the adsorption process on NMAC takes place through these micropores. Thus, processes increase both surface area and pore volume of the prepared sorbent material, which leads to superior adsorption properties. Accordingly, to improve the oleophilic behavior of water hyacinth sorbent material, the extraction of activated carbon from the water hyacinth plant was carried out. Figure 1 represents the oil sorption affinity and water pickup of the prepared NMAC driven from both raw and chemically modified water hyacinth. It was observed that the NMAC produced using zinc chloride activation has a higher oil sorption affinity compared with the phosphoric acid-activated. This is maybe returned to the phosphoric acid increases the ash content of the resulting activated carbon, consequently the carbon content decreases. On the other hand, zinc chloride increases the produced NMAC porosity. Thus, phosphoric acid is not a good activating agent compared with zinc chloride^12^. Moreover, the carbonization and magnetization processes, decrease the water sorption affinity to zero value. Thus, the NMAC prepared from water hyacinth gives more hydrophobic and oleophilic behavior compared with raw water hyacinth and their chemically modified sorbent materials. Accordingly, the water hyacinth carbonization and magnetization process may be considered the best development process to produce good NMAC sorbent material characterized by its high oil sorption affinity and no water pickup.

### Characterization of nano-magnetic activated carbon

The nano-magnetic activated carbon produced after carbonization and magnetization modified water hyacinth roots under heating represents the most efficient prepared sorbent material for oil removal. Accordingly, the properties of this NMAC will be compared with that of the raw water hyacinth segments, to illustrate the effect of carbonization and magnetization process on modifying and improving the internal structural characteristics of the prepared sorbent material.

### X-ray diffraction (XRD)

**Figure 2:**
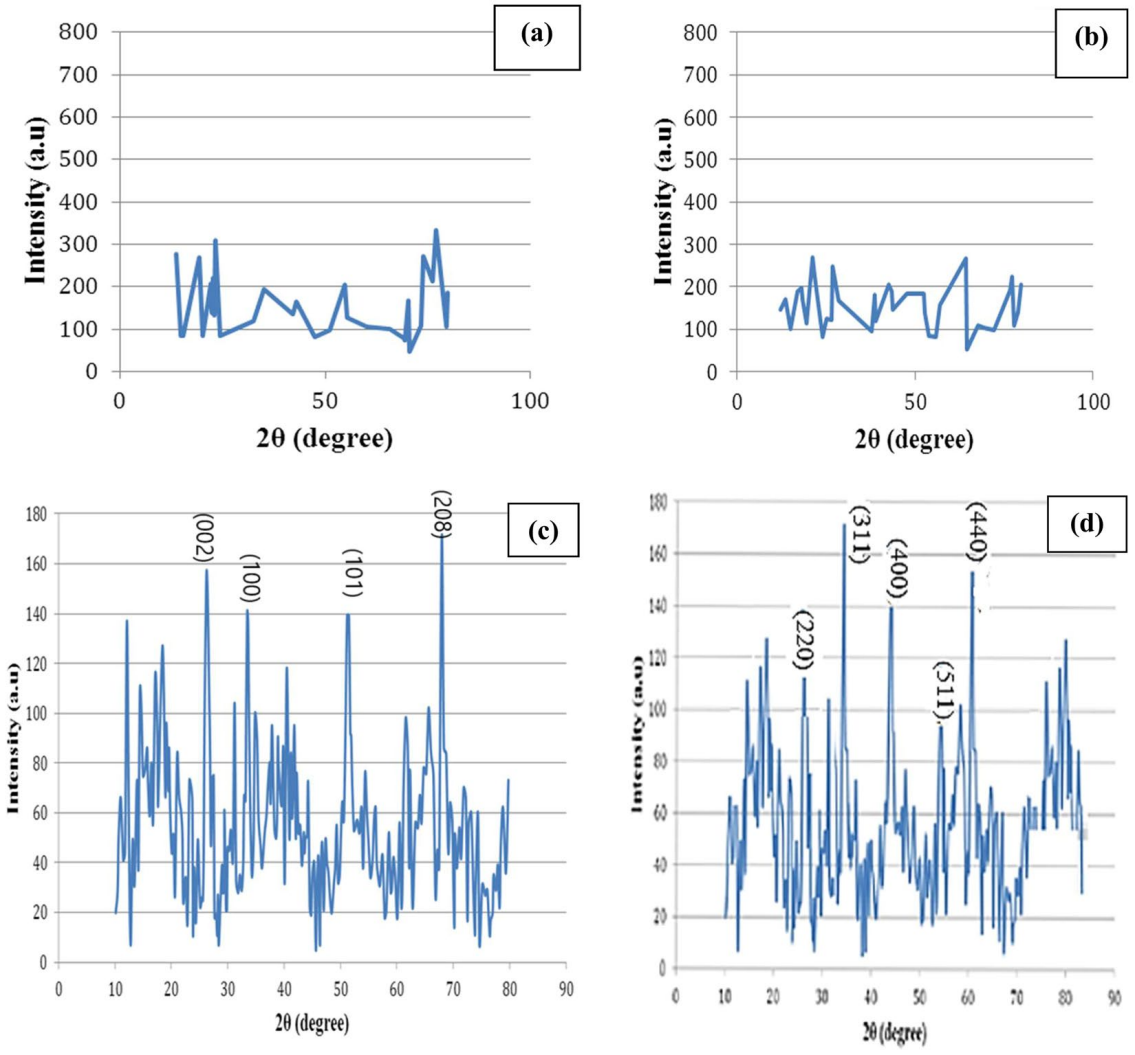
X-ray diffraction pattern (XRD) of the two raw water hyacinth segments, raw shoots and roots and the prepared activated carbon: (**a**) shoots segment, (**b**) roots segments, (**c**) activated carbon product, (**d**) magnetic activated carbon nano-hybrid.

Figure 2 indicates the (XRD) patterns of the two raw water hyacinth segments (shoots and roots), activated carbon and the nano-magnetic activated carbon. It can be concluded that both raw shoots and roots water hyacinth have semi-crystalline structural, where broad peaks were obtained instead of sharp peaks indicating that the samples were poorly crystalline^13^. On the other hand, the produced activated carbon and NMAC seem to have very sharp peaks which indicate that the activation and magnetization processes are accompanied by strong structural changes^14^. In that concern, comparing these patterns is accomplished by using the crystallinity index measurement. The crystallinity of cellulose is calculated according to the empirical method for native cellulose as^15^:

1 $${\rm{CrI}}\,( \% )=(({{\rm{I}}}_{002}\mbox{--}{{\rm{I}}}_{18}^\circ )/{{\rm{I}}}_{002})$$

where CrI is the crystalline index, I_002_ is the maximum intensity of lattice diffraction (002), and I_18_° is intensity diffraction at 18°, 2θ degrees.

XRD profile indicates that the crystallinity index of the two raw water hyacinth segments, raw shoots and roots are 26.4% and 18.7%, respectively and that of the produced activated carbon is 60.8%. Moreover, the crystallinity index of NMAC is higher compared with the other parent samples. This noticeable increase in the crystallinity index of the NMAC sample after the carbonization process may be regarded to more amorphous cellulose breaks down under the activation conditions of adding zinc chloride before carbonization and the heating that takes place during carbonization step at 350–400 °C for two hours. Moreover, the removal of non-crystalline xylan and lignin from raw water hyacinth by the activation process would result in an increase in the crystalline cellulose content of the activated carbon^15^.

### Morphological characterization (SEM)

**Figure 3:**
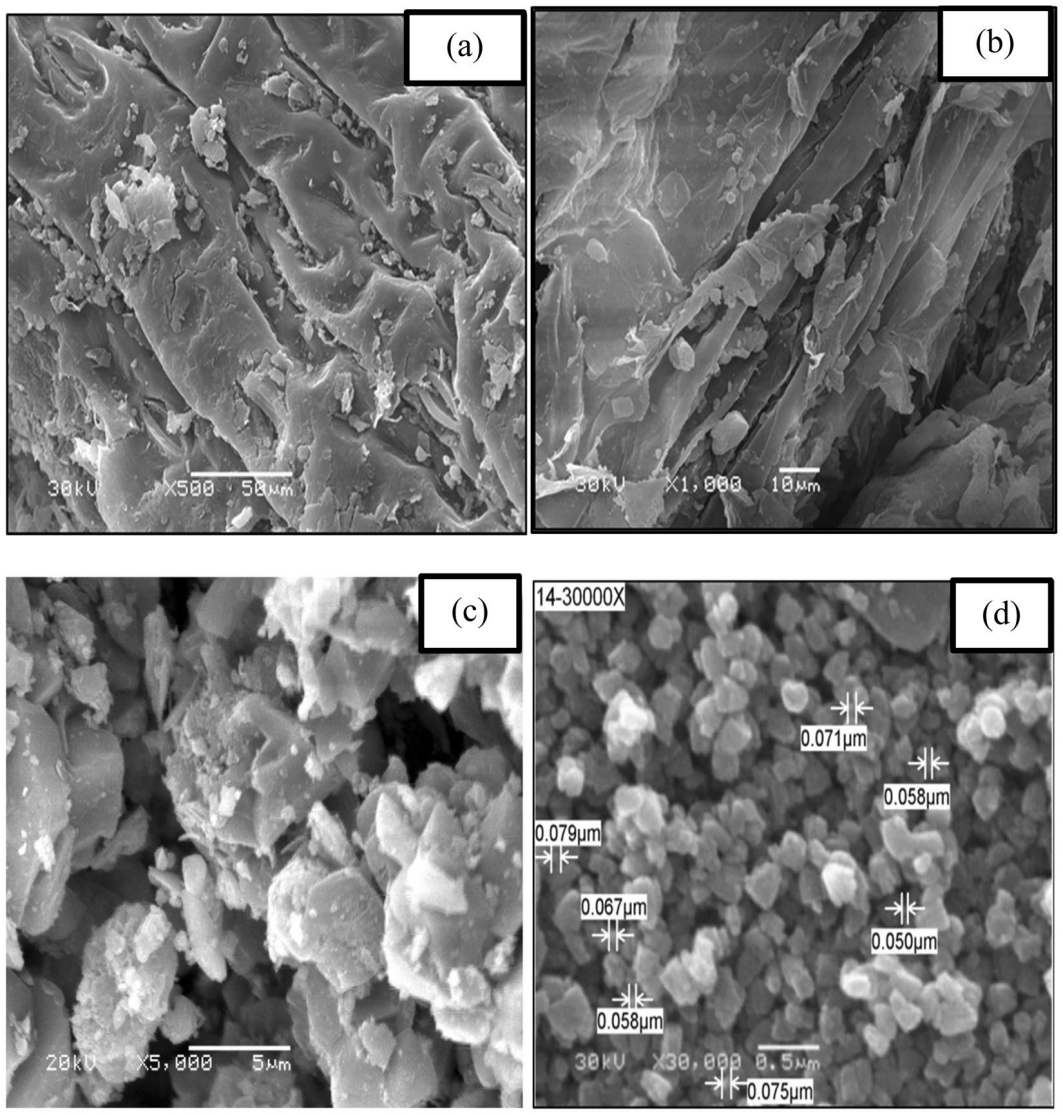
SEM micrographs of surface imaging (**a**) shoots segment of water hyacinth, (**b**) roots segment of water hyacinth, (**c**) prepared activated carbon, (**d**) magnetite immobilized activated carbon.

The morphological structures of natural raw water hyacinth, activated carbon and the NMAC product were compared using SEM to determine changes resulted after the activation and magnetization processes. SEM examination (Fig. 3) revealed that the surface morphology of NMAC is different from that its parents of natural water hyacinth raw and activated carbon, which exhibited wide layer accumulations with porous construction. After carbonization and magnetization processes, the fibers were aggregated and shortened bundles containing big number fibers and some circuitous accumulations of carbon fibers were appeared. Due to diversity caused by the carbonization process of water hyacinth, the pore areas of AC contains smaller pores among the natural fibers whoever the pores become larger among the bundles of fibers. Moreover, it’s observed that the outer surface of prepared activated carbon attain large pores^16,17^. These cavities may resulted from ZnCL_2_ evaporation during the carbonization process^18^.

The surface texture of the most efficient prepared NMAC product was illustrated in Fig. 3d. It is evident that the carbon particles have an aggregation of a very small particles, which don’t have a uniform, fixed size and shape. The particles of different shapes are contained many kinks and steps on the outer surface^19^. It’s clear that the exterior’s surface of the prepared NMAC was converted from micro-scale in the case of raw water hyacinth into nanoscale for the NMAC. According to this micrograph, the average crystallite size of the prepared NMAC was 65 nm. Accordingly, carbonization followed by magnetization processes of raw water hyacinth produces NMAC. These highly microporous structures of nanoscale NMAC can provide the maximum number of possible loading active sites for oil sorption, which results in increasing the oil adsorption capacities of producing NMAC^20^.

### Thermal gravimetric analysis (TGA)

**Figure 4:**
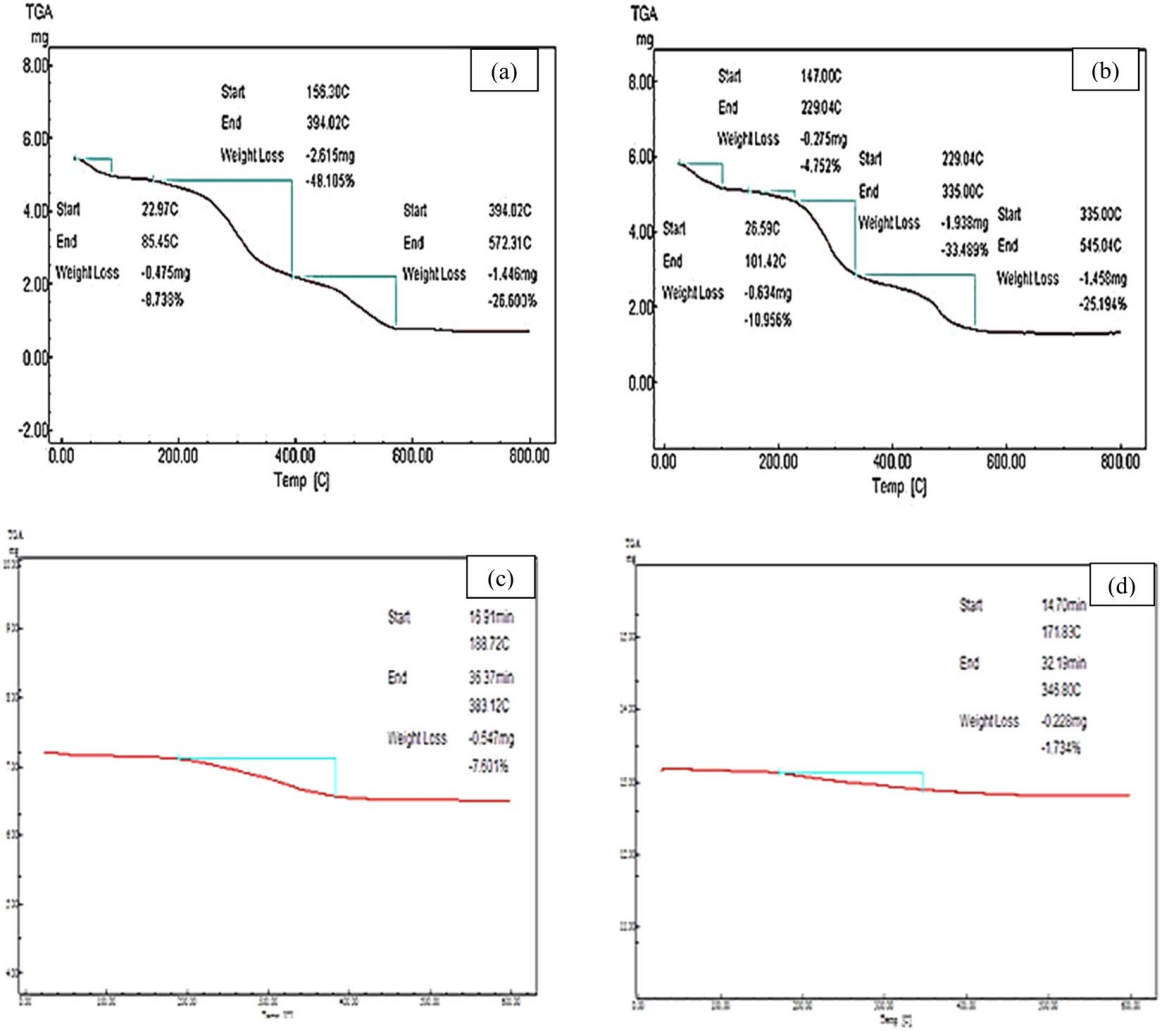
Thermal gravimetric analysis of two raw water hyacinth segments, raw shoots and roots: (**a**) shoots segment, (**b**) roots segment, (**c**) prepared Nano-activated carbon, (**d**) magnetic activated carbon nano-hybrid material.

The water hyacinth plant was examined for its chemical composition, cellulose, hemicellulose, ash and lignin by using TGA analysis. Three major steps were noticed in the thermal decomposition for water hyacinth samples accordingly in Fig. 4. The first degradation step from 20 °C until 100 °C represented the moisture evaporation. The degradation process of cellulose and hemicellulose is the major step in biomass pyrolysis which initiates above 150 °C and ends around 400 °C and it’s expected to lose the most weight of water hyacinth. There are two detectable degradation steps that were noticed around 250 °C and 340 °C. The first at a lower temperature at 250 °C refers to the decomposition of hemicellulose, while the second at a higher temperature at 340 °C represents the cellulose degradation. Further decomposition at 394 °C to 572.31 °C is attributed to lignin degradation^21^. Finally, it can be noticed that there is a variation in boundary temperatures of these stages between shoots and root segments, which is returned to the variation in the composition percentage of these two different plant segments. From the previous thermal analysis, it was revealed a strong agreement between the raw water hyacinth structures, where both segments showed low thermal stability properties.

Furthermore, the thermal stability of both prepared NA carbon and NMAC was explored from Fig. 4. This figure exhibits onset temperatures only at an average temperature of 350 °C for both activated carbon and NMAC. It was indicated that the NMAC attains the highest comparatively thermal stability compared with prepared activated carbon. Where, NMAC recorded only 1.73%wt loss at 346.8 °C compared with 7.6% wt loss at 383.12 °C for prepared activated carbon. This implies that the prepared NMAC has higher thermal stability compared with activated carbon. These results may be owed to loss of carbon dioxide and carbon monoxide from the prepared activated carbon, which may remain unburned or partially burned during the pyrolysis activation process^22^. Hence, comparing this thermo-gravimetric analysis with the two raw water hyacinth segments and prepared activated carbon, NMAC showed the best thermal stability material. So, it can be deduced that the carbonization and magnetization processes improve the thermal stability of the produced adsorbent material.

### Vibrating sample magnetometer (VSM)

**Figure 5:**
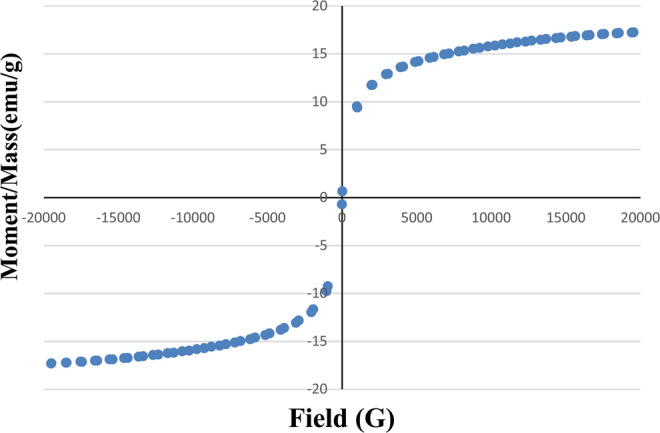
Magnetization curve of the nano-magnetic activated carbon material.

Magnetic characteristics were investigated for the prepared NMAC in Fig. 5 which shows the magnetization curve of the prepared NMAC at room temperature. The hysteresis loop with S-like shape of the prepared NMAC was recorded. As shown in Fig. 5, the material is a typical superparamagnetic. It was observed that the completely saturation moment per unit mass for the NMAC was 17.2587 emu/g. These results agreed with many previous studies stated that the immobilized magnetite nanoparticles show superparamagnetic characteristics when they get smaller than the critical size of the magnetic range size^23,24^.

### BET Surface Area

**Figure 6:**
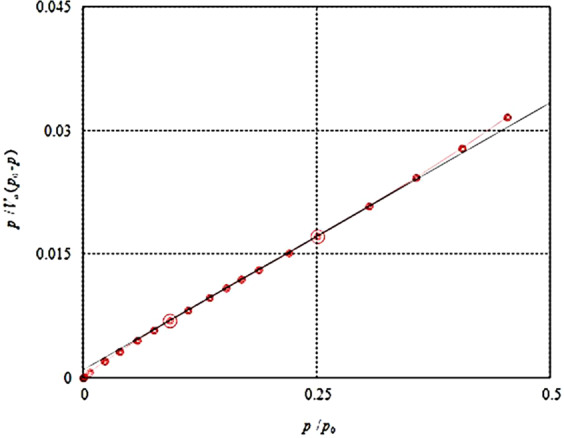
Nitrogen adsorption-desorption isotherms of the nano-magnetic activated carbon material.

As shown in Fig. 6, the N_2_ adsorption-desorption isotherms of NMAC were examined and the BET surface area was of the prepared NMAC material was recorded as 66.27 m^2^/g. This surface area result is relatively high for materials adsorption. Also, the fabricated NMAC was characterized by its high total pore volume of 0.25 cm^3^/g compared with 0.183 cm^3^/g for NAC from raw Egyptian coal source called Maghara coal^25^. Furthermore, the average pore diameter of the fabricated NMAC was assigned as 15.29 nm. According to these results, the fabricated NMAC achieved significantly high surface area and porosity, which are very important for different potential applications^25^.

### Adsorption technique for oil spills cleanup process

In order to set the optimal operating parameters dominating the oil adsorption process using the most efficient prepared sorbent material (NMAC), the effect of adsorbent dose, sorption time, agitation speed, initial oil concentration and temperature were investigated.

### Influence of adsorption time

**Figure 7:**
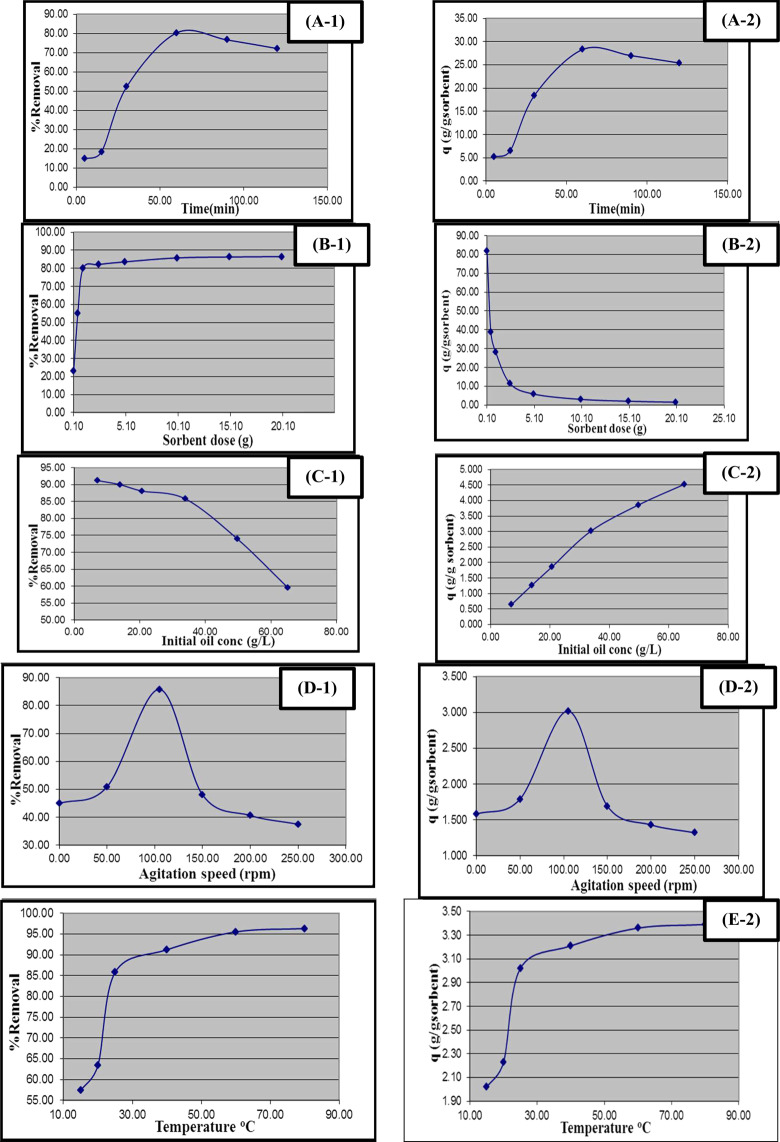
Influence of oil spill removal processing parameters onto both percentage oil removal and oil adsorption capacity using nano-magnetic activated carbon material: (A) sorption time, (B) sorption dosage, (**c**) initial oil concentration, (**d**) agitation speed, (**e**) temperature.

The adsorption contact time represents an essential factor in the oil adsorption control processes onto the fabricated NMAC^26^. Figure 7-A investigates the effect of adsorption time on the percentage of oil removal and its adsorption affinity onto the prepared NMAC sorbent material. It’s observed that oil adsorbed removal percentage onto the NMAC was increased with sorption time increasing until it reaches a maximum value at 60 min and then decreased to nearly constant values irrespective of the sorption time. So that, 60 min is considered as the optimum equilibrium time for the process of adsorption. The percentage rate of oil removal is higher at the start of the adsorption process due to the presence of a high surface area of NMAC for the adsorption of oil. For more explanation, there is a fast propagation onto the exterior NMAC surface. This process is followed by a very slow diffusion into the inner-particles to reach the equilibrium state at 60 min. After equilibrium, the transferring rate of oil to the active sides of the adsorbent material was decreased. The two phases sorption mechanisms with the first quantitatively predominated and fast and the second quantitatively insignificant and slower resulted in increasing the adsorbed oil up to the maximum value attained at the equilibrium and decreased after the equilibrium time^23,27^.

### Influence of NMAC adsorbent dosage

Adsorbent dosage has a strong impact on the adsorbent affinity at specified initial pollutant concentration^23^. The effect of the adsorbent dosage on the percentage removal of oil and the adsorption affinity onto the synthetic NMAC were investigated at Fig. 7-B1. It was indicated that by increasing the adsorbent dose, the percentage of oil removal is increased until it attains near higher value at 1 g of the sorbent material. As the sorbent dosage improved the oil adsorption maintains nearly at a constant value until sorbent dosage increased up to 10 g that slight enhancement the percentage oil removal. This may be attributed to the availability of more adsorption activates areas onto the adsorbent material at constant oil concentration. So, the sorbent amount of 1 g represents the optimum dosage from NMAC for the oil spill cleanup process.

On the other hand, Fig. 7-B2 shows the effect of the adsorbent dosage, since when it increase from 0.1 to 20 g, the oil uptake affinity decreases from 82.01 to 1.52 g gas oil/g. Many suggested reasons can contribute to this behavior. This decrease in oil adsorption affinity is principally because of the non-saturation state of the adsorption pores at adsorbent material. On the contrary, it may be due to the behavior of the particle interactive, such as aggregation which is resulting from the high dosage of the adsorbent. This aggregation causes decreasing the total external surface area of the adsorbent material and therefore increase in the oil diffusion path length^24,27^.

### Influence of the thickness of oil film (Initial oil concentration)

The initial concentration of the removed oil waste is represented in the case of the oil spills by the initial oil film thickness, which can be then converted into a concentration by using the whole volume of the polluted solution (water and oil). Figure 7-C(1, 2) investigate the effect of the initial concentration of oil on the percentage removal of oil as well as the adsorption affinity of oil onto the prepared NMAC.

Figure 7-C1 shows that the percentage of removal is inversely proportional to the oil concentration. It’s also observed that the removal of oil decreases from 91.2% to 59.5% with increasing the oil concentration from 6.98 to 65.19 g/L which represents the improvement of oil film thickness from 1 and 10 mm, respectively. This behavior demonstrates that there was a reduction in instant oil adsorption, due to the lack of available effective areas of the adsorbent material which required for the high initial concentration of oil. This may be owed to the improvement of oil surface adsorption onto NMAC that significantly increases the oil agglomerates onto NMAC particles that hindered the oil adsorption process^28^.

However, as indicated in Fig. 7-C2, the increase in initial oil concentration is associated with the increment in the adsorption affinity. Where, the increment at initial oil concentration from 6.89 to 65.19 g/L, the adsorption affinity of oil onto the NMAC improved from 0.642 to 4.52 g gas oil/g sorbent, this designates that the initial oil concentration has a significant role in the adsorption affinity of NMAC. This enhancement at oil adsorption may be returned t the reduction in uptake resistance of the oil from the waste solution^29^.

### Influence of mixing rate

The rate of mixing represents a vital factor in the oil adsorption phenomena, where, it has a thoughtful effect on both the oil distribution at the bulk solution and the establishment of the exterior boundary film. The influence of mixing rate on the percentage oil removal and the adsorption affinity of oil onto the prepared NMAC was examined in the range of 0–250 rpm of the agitation speed. Figure 7-D illustrates that the percentage oil removal and the uptake affinity were affected by the mixing rate for values between 0 to 105 rpm. This result approving the significant role of the external diffusion onto the sorption kinetics of oil adsorption process^30^. Also, increasing the rate of mixing from 105 to 250 rpm that decreasing the percentage removal from 85.8% to 37.5% and the uptake oil’s affinity decreased from 3.02 to 1.32 g oil/ g sorbent.

This decline at oil removal and uptake affinity may be returned to the increment at the desorption tendency of oil molecules due to stable film formation at the surface of the adsorbent. This propensity for the oil desorption process may be returned to the high rate of agitation that required more input of energy which developed a high shear force that breaks the bonds between the oil molecules and the adsorbent material^31^. Accordingly, the mixing rate of 105 rpm is adequate to be confirmed that all the available NMAC binding sites at the surface are active for oil adsorption.

### Influence of temperature

The dependence of the percentage oil removal and the oil adsorption affinity onto the prepared NMAC have been investigated at 15, 20, 40, 60 and 80 °C, as shown in Fig. 7-E. It was evident that the percentage oil removal and the adsorption affinity increase with temperature regarded to the increment of oil diffusion rate to the adsorbent. In addition, the equilibrium affinity of the adsorbent was changed as the adsorbent temperature varied^32^. It is clear from Fig. 7-E that increasing temperature from 15 to 80 °C, the percentage oil removal improved from 57.4 to 96.3% and adsorbed oil amount incremented from 2.02 to 3.39 g oil/g sorbent, indicating that the oil sorption process is endothermic.

### Equilibrium isotherms and adsorption kinetics modeling

Regarding to improve the operation of the oil spill adsorption process using prepared NMAC, it is essential to construct the best suitable relationships for describing the operation at equilibrium. Accordingly, the kinetics of the oil spill process as well as the adsorption at equilibrium using the most efficient prepared NMAC were investigated using different empirical correlation models.

### Isotherm analysis

The adsorption data of oil spills were analyzed using four different isotherm equations including Langmuir, Freundlich, Temkin, and Dubinin-Radushkevich (D-R). The availability of these correlations to describe the oil adsorption process was validated using the correlation coefficients, R^2,33^.

**Figure 8:**
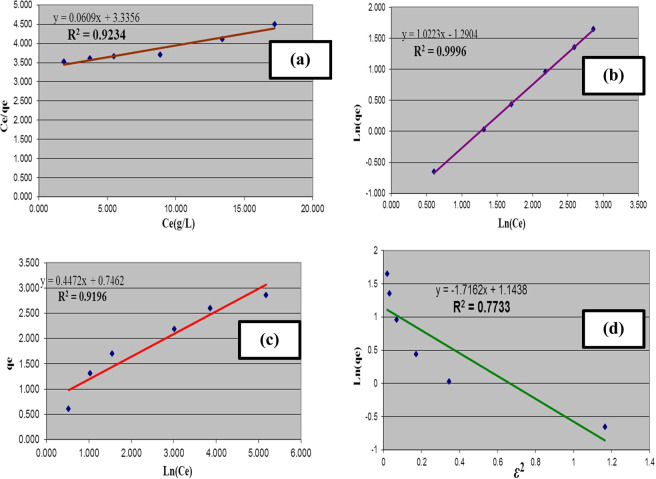
Equilibrium modeling of oil sorption process onto nano-magnetic activated carbon material: (**a**) Langmuir isotherm, (**b**) Freundlich isotherm, (**c**) Temkin isotherm, (**d**) Dubinin-Radushkevich isotherm.

Langmuir isotherm is depended upon the hypothetical suggestion that the surface adsorption process takes place only as monolayer onto the adsorbent material. Moreover, it signifies distribution of the oil molecules at equilibrium between solid and liquid phases. A straight line relationship was obtained when plotting C_e_/q_e_ against C_e_, the slope of this line was assigned as 1/q_m_ and the intercept as 1/q_m_ K_L_^34^. The values of Langmuir constants q_m_ and K_L_ are investigated at Table 1. The essential dimensionless equilibrium parameter of the Langmuir isotherm is defined by^27^.

2 $${{\rm{R}}}_{{\rm{L}}}=1/(1+{{\rm{K}}}_{{\rm{L}}}{{\rm{C}}}_{{\rm{o}}})$$

where K_L_ is the Langmuir constant and C_o_ is the initial oil concentration. The value of R_L_ designates the category of the isotherm. All the values of R_L_ for the different studied initial oil concentrations are listed in Table 1 and are detected to be ranged between 0–1, demonstrating that the oil adsorption process is favorable onto the adsorbent material NMAC. The linear plot of the Langmuir equation is illustrated in Fig. 8-a. The comparatively low value of correlation coefficient R^2^ (0.92) presented that the oil adsorption process onto the prepared NMAC adsorbent may be designated through Langmuir isotherm^35^.

**Table 1:** Estimated parameters for oil adsorption process onto NMAC.

Isotherm	Parameters	Value
Langmuir	q_m_ (mg/g)	30.20
	K_L_ (L/g)	0.018
	R^2^	0.923
Freundlich	K_f_ (g/g (L/g)^1/n^)	0.275
	n_f_	1.080
	R^2^	0.999
Temkin	K_T_ (L/g)	5.305
	R^2^B (J R^2^	0.910
Dubinin–Radushkevich	K‘ (mol^2^/kJ^2^)	1.716
	V’_m_ (g/g)	3.139
	R^2^	0.773
	E (kJ/mol)	0.539

The Freundlich isotherm is an extensively utilized equilibrium model, it is suggested the heterogeneity of the adsorbent surface^36^. The intercept and slope of Freundlich plot were estimated from plotting of ln (q_e_) against ln (C_e_) which are investigated in Table 1 with their correlation coefficient, R^2^. The line intercept, K_f_ gives a rough indication about the adsorption affinity and the slope, n_f_, gives prediction about the effectiveness of adsorption process. Figure 8-b demonstrates the linear plot of Freundlich isotherm. The correlation coefficient is determined as R^2^ = 0.9996, which is agreed with the experimental results of the oil adsorption process. This result evident that the oil adsorption process onto NMAC follows the Freundlich equation. The calculated values of n_f_, were greater than 1 that indicates favorable oil adsorption onto NMAC^37^. The value of Freundlich constant specifies facile oil removal from aqueous solution. Regarding to Freundlich isotherm exhibited the greatest fitting, this supposed that there is a heterogeneity on NMAC surfaces or its pores had as significant role in the oil sorption^34^.

Temkin reflected the influences of indirect relationships between adsorbent and adsorbate on the adsorption isotherm that suggested adsorption is described by a homogeneous binding energies distribution that equal to specific maximum binding energy^37,38^. Plotting of q_e_ versus ln (C_e_) allows estimation of slope and intercept to determine the isotherm constants B and K_T_ respectively. The Temkin model parameters and its correlation coefficient, R^2^ are tabulated in Table 1. Figure 8-c demonstrates the linear plot of Temkin isotherm. Its correlation coefficient value of R^2^ = 0.919 indicating relatively poor fitting to the isotherm equation.

In order to define the single solute sorption isotherms, the D–R isotherm is frequently utilized. The D–R isotherm is more general compared with Langmuir isotherm due to it discards the constant adsorption potential^39^. The constant K‘ represents the average oil sorption free energy per NMAC molecule. This energy may be calculated using the following correlation:

3 $${\rm{E}}={(2{\rm{K}}{\prime} )}^{-0.5}$$

The equilibrium results of oil adsorption onto NMAC were applied by D–R isotherm equation for determination of the sorption process nature whether it is physically or chemically process. Plotting of ln (q_e_) against ε^2^ is illustrated in Fig. 8-d. The correlation coefficient of this plot was equal to 0.77333 that designates that the D–R models are not adequate to describe the experimental results compared with Langmuir, Freundlich and Temkin isotherm models.

The D–R model parameters are tabulated at Table 1. The computed adsorption energy (E < 8 kJ/mol) shows that the oil adsorption process may be classified as physisorption process.

Finally, considering that the oil sorption process obeyed the Langmuir isotherm that gives prediction about the monolayer coverage of oil onto the prepared NMAC surface. However, the adsorption process of oil is considered a complex process, possibly multi-layers may be formed and/or some pores of NMAC may be closed. These expectations of the adsorption mechanism of oil onto the prepared nano-magnetite activated carbon were confirmed through the Freundlich model. Where difference at sorption affinity is predictable with surface coverage^40^. The Freundlich equation produced the largest R^2^ value (0.9996) which indicates that the oil sorption process onto the prepared NMAC best defined using Freundlich equation confirming multi-layers adsorption as expected. Moreover, the Langmuir isotherm model has some degree of applicability to describe the oil sorption process onto the prepared NMAC. Where its correlation coefficient value represents the highest value after Freundlich isotherm. Accordingly, these results suggest that the oil sorption process takes place onto the NMAC as mono-layer coverage and there is some heterogeneity in the oil sorption process onto the synthesized NMAC that covered as multi-layers adsorption. This proposed some heterogeneity on the material surfaces or pores of the prepared NMAC played role in oil sorption.

### Comparable investigation of adsorption capacity of various AC based materials

The adsorption performance of the fabricated NMAC with various previously investigated low-cost activated carbon-based materials was compared based upon mono-layer adsorption capacity (q_m_). Table 2 elucidates the adsorption capacity of fabricated NMAC for oil spills compared with various studied low-cost activated carbons. It was designated that the monolayer adsorption capacity of NMAC is largest compared with other literature studied activated carbon materials 47. Consequently, the fabricated NMAC from water hyacinth roots introduced as an appropriate and promising material for oil spills removal from polluted water.

**Table 2:** Comparable investigation for mono-layer adsorption capacity of prepared NMAC with various fabricated AC materials.

Adsorbent material	q_m_(mg/g)	Reference
NMAC fabricated using water hyacinth roots	30.20	Present study
AC fabricated using Delonix regia pods	24	^44^
AC fabricated using wheat shells	16.56	^45^
AC fabricated using mature leaves of the Neem tree	8.76	^46^
AC fabricated from coconut husk	5.87	^47^

### Kinetics of oil spill process

The investigation of oil sorption kinetics onto the prepared NMAC is desirable to offer specific knowledge about the mechanism of the oil adsorption process. Four different kinetic models, pseudo first-order, pseudo second-order, Elovich and intraparticle diffusion kinetic models were designated to define their applicability for description of the oil sorption process^27^.

**Figure 9:**
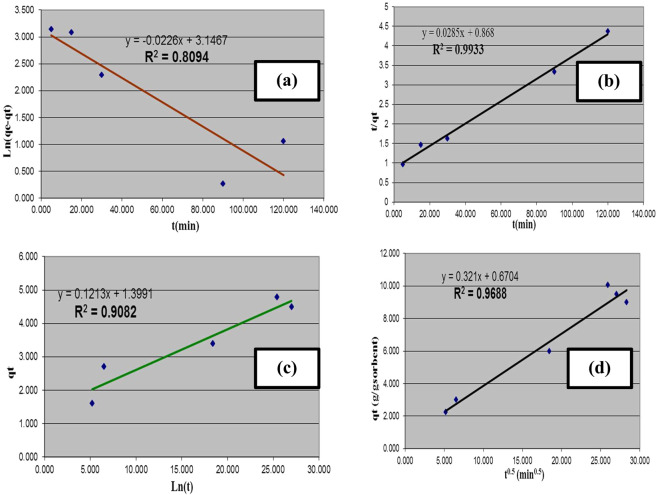
Kinetics of oil sorption process onto nano-magnetic activated carbon material: (**a**) Pseudo- first-order, (**b**) Pseudo- second-order, (**c**) Simple Elovich, (**d**) Intraparticle diffusion.

Pseudo first-order kinetic model was described as follows:

4 $${{\rm{dq}}}_{{\rm{t}}}/{{\rm{d}}}_{{\rm{t}}}={{\rm{K}}}_{1}\,({{\rm{q}}}_{{\rm{e}}}-{{\rm{q}}}_{{\rm{t}}})$$

where q_e_ and q_t_ are oil quantities (g/g) adsorbed on the adsorbent at equilibrium and at time t, respectively, and K_1_ is the first-order reaction rate constant (min^−1^). Figure 9-a illustrates the linear plot of ln (q_e_ − q_t_) against t, from this figure both K_1_ and q_e_ were calculated from the slope and intercept respectively for oil spill process onto prepared NMAC that were tabulated at Table 3. It was evident from the table that the correlation coefficient for the linear fitting is not high enough (R^2^ = 0.809). Moreover, the calculated values of q_e_ using first-order equation were not similar to that recorded from experimental work as evident from Table 3. Consequently, it may be concluded that the first-order model hasn’t good agreement with the oil spill process.

**Table 3:** Kinetic parameters for adsorption of oil onto prepared NMAC.

Kinetic model	Parameters	value
Pseudo first-order	(q_e_)_cal_(g/g)	23.26
	(q_e_)_exp_ (g/g)	28.31
	k_1_ (min^−1^)	0.0226
	R^2^	0.809
Pseudo second-order	(q_e_)_cal_ (g/g)	25.81
	(q_e_)_exp_ (g/g)	28.31
	k_2_(g/g.min)	1.74×10^−3^
	R^2^	0.9933
Elovich model	α(g/g.min)	1.3991
	β (g/g)	0.1213
	R^2^	0.908
Intraparticle diffusion model	k_id_ (g/g.min)	0.321
	I	0.6704
	R^2^	0.9688

The pseudo second-order rate model depend upon the sorption equilibrium affinity and formulated as follows^27^:

5 $$1/({{\rm{q}}}_{{\rm{e}}}-{{\rm{q}}}_{{\rm{t}}})=1/{{\rm{q}}}_{{\rm{e}}}+{{\rm{k}}}_{2}{\rm{t}}$$

where k_2_ is the rate constant of the pseudo second-order sorption (g/g. min). Equation (5) represents the linear form pseudo second-order rate model. Figure 9-b showed the linear plot of t/q_t_ versus t for the oil spill process, the equation constants of k_2_ and q_e_ were calculated using the intercept and slope respectively and tabulated at Table 3. Furthermore, the calculated values of q_e_ using Eq. (5) are tabulated at Table 3. It was indicated from this table that the correlation coefficient R^2^ value recorded the largest value equal of 0.9923. This result elucidated that the oil spill process onto NMAC is dominated by the pseudo second-order adsorption mechanism. Furthermore, the estimated values of q_e_ from pseudo second-order equation were almost similar to that recorded from the experimental data. Consequently, the second-order rate equation fits the data most reasonably.

Elovich model is representative for explaining the chemisorption kinetics processes. Figure 9-c indicated the linear plot of q_t_ against ln t. The estimated Elovich parameters from the slope and intercept of the simple Elovich plot are listed in Table 3. Where, β is suggestive of for the available sites for oil adsorption onto NMAC, while α is representative for adsorption quantity. It was showed from Table 3 that the correlation coefficient value is reasonably low (R^2^ = 0.9082) indicated that the Elovich model is not adequate to describe the experimental data. This result proposed that the oil spill process onto NMAC may occurs through physical interaction process.

Finally, in order to determine the nature of rate-limiting step in the oil spill process, intraparticle diffusion model was investigated through the following Eq. (6).

6 $${{\rm{q}}}_{{\rm{tt}}}={{\rm{k}}}_{{\rm{id}}}{{\rm{t}}}^{0.5}+{\rm{I}}$$

where it gives prediction about the boundary layer thickness. Figure 9-d illustrated the linear plot of q_t_ versus t^0.5^ that used for estimation the equation constants k_id_ and I from the slope and intercept respectively. Table 3 indicated a low value of R^2^ that equals 0.9688 demonstrating that the oil spill process can be described by the intraparticle diffusion model. Additionally, the liner plot did not intersect with the origin, these results evident that the intraparticle diffusion was not the only rate-limiting step.

Comparing the R^2^ values of the previous three studied models, it was indicated that the oil spill process can be well described using pseudo second-order model. The main adsorption mechanism proposed to take place for oil spill using NMAC is possibly physico-sorption, including van der Walls forces between the sorbate and sorbent^35^. Moreover, the great value of R^2^ for the linearity of the diffusion model supposed that the oil spill process is principally controlled by the intraparticle diffusion.

### Adsorption thermodynamics

The thermodynamic parameters of the oil spill process were investigated to determine the nature of the adsorption process. The standard Gibbs free energy (ΔG°), standard enthalpy (ΔH°) and standard entropy (ΔS°) related to the adsorption process were calculated using the following equations^34^:

7 $$\Delta {\rm{G}}^\circ =-\,{\rm{RT}}\,\mathrm{ln}\,{{\rm{K}}}_{{\rm{c}}}$$

8 $${{\rm{Ink}}}_{{\rm{c}}}=\frac{{\Delta {\rm{S}}}^{0}}{R}-\frac{{\Delta {\rm{H}}}^{0}}{{\rm{RT}}}$$

where R is the gas constant equal to 8.314 J.mol^−1^ K^−1^ and T is the absolute temperature in Kelvin. K_c_ (q_e_/C_e_) is a constant of equilibrium at different temperatures. The linear plotting of lnK_c_ versus 1000/T utilized to estimate ΔH° and ΔS° from the slope and intercept respectively as tabulated in Table 4. The negative values of ΔG° indicated that the sorption of oil spills onto NMAC was a spontaneous process. Besides, the decline at ΔG° (−4.87 to −10.44 KJmol^−1^) as temperature improved confirmed favorable adsorption at elevated temperatures. The positive value of ΔH° (23.49 KJmol^−1^) demonstrated the endothermic nature of oil spills’ adsorption onto the fabricated NMAC. The positive value of ΔS° (96.02 J mol^−1^ K^−1^) revealed that the degree of haphazardness improved at the solid/liquid interface through the oil sorption process^27^.

**Table 4:** Thermodynamic parameters for oil sorption onto nano-magnetic activaited carbon.

Temp.(K)	1000/T	K_c_	lnK_c_	ΔG^0^ (kJ·mol^−1^)	ΔH^0^ (kJ·mol^−1^)	ΔS^0^ (J·mol^−1^·K^−1^)
298	3.356	7.163	1.969	−4.878	23.495	96.027
313	3.195	10.547	2.356	−6.119		
328	3.049	26.933	3.293	−8.983		
343	2.915	30.056	3.403	−9.706		
358	2.793	33.364	3.508	−10.44		

## Conclusion

This study focused on the cleanup of oil spills from contaminated wastewater using an innovative magnetic sorbent material driven from the water hyacinth. The chemical and thermal treatment conditions of water hyacinth implied that the root segment of the water hyacinth plant that considered the submerged part of the plant has better oil adsorption behavior compared with the shoots segment. The characteristics of the two-different raw water hyacinth segments (shoots and roots), prepared nano-activated carbon and NMAC indicated that the NMAC sample has the highest organized crystalline structure, noticeable development of pores and thermal stability. Also, NMAC sample recorded the completely saturation moment per unit mass value at 17.2587 emu/g. The increment at the solution temperature and NMAC dosage enhance the percentage oil removal. The equilibrium isotherm model’s applicability of the oil sorption process obeys the order of Freundlich> Langmuir> Dubinin-Radushkevich, therefore it can have suggested that the oil sorption takes place onto NMAC as mono-layer accompanied with some oil sorption heterogeneity on the NMAC material surfaces or through its pores. The kinetic investigation of the oil spill process onto NMAC designated that the adsorption kinetics may be described by pseudo second-order model conjugated with intraparticle diffusion model. The proposed main mechanism for oil spill process onto NMAC is physico-sorption including van der Waals forces between oil and the prepared NMAC.

## Materials and Methods

### Materials

The raw water hyacinth was collected from Itay El-Baroud Drainage, Al-Buhayrah governorate, Egypt. The synthetic liquid waste oil used is the gas oil suspended in artificial sea water, this fuel oil obtained from petroleum distillation. Pad from Non-woven polypropylene fiber was utilized as a supporting matrix for the different powdered sorbent materials derived from water hyacinth to determine their behavior in oil spills removal process. This pad has no affinity for oil sorption. It has an average thickness of 0.65 mm, tensile strength of 6.25 N/cm^2^ and elongations 32.2%. All chemicals utilized without purification and they are of analytical grade such as hexane, hydrochloric acid, sodium hydroxide, phosphoric acid and zinc chloride. Hexane was used as a solvent which helps the gas oil to be extracted during the press stage of the test after the sorption process.

### Preparation of adsorbent materials

The collected water hyacinth plant was carefully washed with distilled water to eliminate dust, fungus and other foreign materials. After washing it was dried in the electric oven at 40 °C till complete dryness. The two segments of the plant (shoot and root) were separated. Size reduction of these two different segments of the water hyacinth was done in a specified grinding machine to reduce the plant size and make it capable of being sieved to the required particle size (1 mm).

### Raw Water Hyacinth as an Adsorbent Material

The two water hyacinth segments shoot, and root powdered materials with 1 mm average particle size were used as raw natural sorbent materials for oil spill cleanup. The oil sorption affinity of the two different water hyacinth segments and water pickup were determined using batch technique.

### Chemical modification process of raw water hyacinth segments

Chemical modification of the two different water hyacinth segments was accomplished using both alkali treatment (NaOH, 1 M) and acidic treatment (HCl, 1 M), to improve their active surface area and induce a new exchangeable (H^+^) and (OH^-^) ions inside their structures. The alkaline and acidic activation processes were carried out with and without heating. The chemical modification temperature was occurring at 60 °C. Chemical modification of raw water hyacinth was carried out in four steps: (i) Five grams of powder material (roots or shoots) with a particle size of 1 mm were added to 50 ml from either 1 M HCl solution for the acidic treatment or 1 M NaOH solution for the alkaline treatment. (ii) The slurry was shaken at agitation speed of 150 cycles/min for 4 hours at the room temperature or under heating at 60 °C. (iii) The slurry was filtered after completeness the reaction time and dried at 60 °C.

### Preparation of nano-magnetic activated carbon from water hyacinth

The purpose of the water hyacinth carbonization process was to improve the oleophilic and hydrophobic characteristics of water hyacinth and to convert its cellulose contents into activated carbon. To improve the porosity of the produced activated carbon, chemical activation process has been used for the powdered materials before the carbonization process. The two different powdered segments of water hyacinth before and after chemical modification process were chemically activated before the carbonization process. Phosphoric acid (H_3_PO_4_) and zinc chloride (ZnCL_2_) are utilized to treat the parent adsorbent materials prior to carbonization process. The main feature of all chemicals utilized at the chemical modification process that they act as dehydrating reagents that cause pyrolitic decomposition and prevent tar formation improving carbon production. The preparation of activated carbon from water hyacinth was carried out in seven stages as follows (i) 5 g of powder sorbent materials were added to both phosphoric acid and zinc chloride solutions with concentrations. (ii) The mixtures were mixed at 60 °C for 6 hours by agitation at 200 cycles/ min using the shaking water bath. (iii) The resulting homogeneous slurries were filtered and washed with distilled water and hot distilled water to remove the un-reacted chemical compounds. (iv) The filtered cakes were dried at 100 °C for 24 hours. (v) The produced samples were allocated at vertical tubes with a length of 12.5 cm and inner diameter of 2.5 cm that includes a small top hole for gas ventilation. (vi) The tubes were heated in a muffle furnace over temperature range 350–400 °C for two hours. (vii) Finally, the activated carbon samples were chilled to the room temperature in presence of nitrogen gas for one day. Consequently, homogeneous suspension from the prepared sample was prepared through suspension a specific weight from activated carbon of 1.5 g using the ultrasonic homogenizer (Sonics Vibra-Cell VCX 500, USA) at 200 ml mixed solution. The mixed solution composed from two iron salts of iron (III) chloride and iron (II) sulphate with molar ratio 2:1 respectively. In order to immobilized magnetite onto prepared activated carbon, 5 M of NaOH solutions was supplementary drop wise to the previously prepared suspension at 70 °C and preserved for 30 min below continuous stirring to produce black precipitate^27^. The obtained powder was washed numerous times with distilled water and ethanol, then the powder was separated using centrifugation force at 4000 rpm. Finally, NMAC was dried at 70 °C overnight.

### Characterization of the adsorbent material

The physicochemical properties of the most efficient prepared NMAC were examined using different characterization techniques of X-ray Diffraction (XRD), Scanning Electron Microscope (SEM), Thermal Gravimetric Analysis (TGA), and Vibrating Sample Magnetometer (VSM). X-ray diffractometry was performed by X-ray diffractometer with CuK-alpha radiation beam (λ = 0.154060 nm) to determine the structure of the raw water hyacinth and the most efficient prepared NMAC. Data were collected between 10° and 80° in 2θ.

The water hyacinth and prepared NMAC were stocked over a holder of the scanning electron microscope (SEM) (JEOL JSM 6360LA, Japan). Then they were gold-sputtered before the examination. The structure and particle diameter of prepared samples were determined at diverse magnifications.

Thermogravimetric analysis (TGA) method was employed to examine the thermal degradation behavior of the samples. The thermal stability of two raw water hyacinth segments and the most efficient, prepared NMAC was evaluated using Thermo-Gravimetric Analyzer (Shimadzu TGA-50, Japan) and platinum sample holder for heating samples. Approximately 5–10 mg of selected sample was pyrolysis at the temperature increment rate 10 °C/min using TGA unit.The magnetic properties of the fabricated NMAC were investigated at room temperature up to a maximum magnetic field of 900 Tesla using a vibrating sample magnetometer (VSM) (Dexing, Model: 250, Lake Zurich, IL, USA).

Finally, The Brunauer-Emmet-Teller (BET) surface area was investigated using BEL Japan, Inc. by measurement of the N_2_ adsorption-desorption isotherms. Before measurements, all samples were degassed under vacuum at 25 °C for 12 h.

### Adsorption technique for oil spills cleanup process

In this work, adsorption of oil spill has been studied using batch technique. Primarily, experimental procedures were carried out on the two different segments of raw water hyacinth (shoots and roots) to study their oil sorption affinity and water pickup under simulated field conditions. Then, they were chemically modified using alkaline or acidic modification process. The produced powdered materials either after the chemical modification process or after the carbonization and magnetization process were tested to determine their oil sorption capacities and water pickup after the oil spill process. The oil spill sorption properties of all prepared materials were compared to identify the most efficient material that records the highest oil sorption affinity and lowest water pickup.

Oil spills sorption affinity determination under simulated field conditions (dynamic test) was accomplished through these following steps^41^: (i) 500 ml of artificial sea water (3.5% NaCl) was placed in a one-liter glass beaker (cell). (ii) A certain amount of oil (40 ml) was added to the beaker to give 5.00 mm oil slick. (iii) The bath temperature was kept constant at 25 ± 1 °C using the thermostatic water bath. (iv) Specific weight of the sorbent material was placed on a polypropylene pad with surface area of (5 × 5) cm^2^. (vi) After certain shaking period, the wetted sorbent material was then removed with the net and let to drain over the beaker for 5 min. (vii) The sample weight was determined and the oil sorption affinity was calculated. (viii) The sample and any residual that remained in the watch glass were transferred to the piston to extract the oil. (x) The water content was determined by the centrifuge technique described in ASTM D-1696-91^42,43^. (xi) The amount of oil that sorbed by either the water hyacinth segments or prepared NMAC material was determined. This test is designed to simulate field conditions and to study the oleophilic and the hydrophobic behavior of the final form of the prepared sorbent materials. The removal percentage of oil was calculated according to the following equation:

9 $$ \% \,{\rm{oil}}\,{\rm{removal}}=(({{\rm{C}}}_{0}-{\rm{C}})/{{\rm{C}}}_{0}\,\ast \,100)$$

where C_0_ and C (both in g/L), are the initial concentration and the concentration at any time, respectively.

Moreover, the removal affinity per gram NMAC was calculated by:

10 $${\rm{q}}({\rm{g}}\,{\rm{gas}}\,{\rm{oil}}/{\rm{g}}\,{\rm{sorbent}})=({{\rm{C}}}_{0}-{\rm{C}})\,\ast \,({\rm{V}}/{\rm{M}})$$

where V is the solution volume (Liter) and M is the adsorbent amount (g).

The different processing parameters that affected by the oil sorption process and the sorption affinity of the prepared NMAC driven from water hyacinth were studied. The influence of wide range variation of the contact time (5–120 min), NMAC dosage (0.1–20 g/L), Oil film thickness (1–10 mm), Agitation speed (0–250 cycle/min) and solution temperature (15–80 °C) onto the oil sorption process was explored. All oil adsorption experiments were performed in triplicate and the average value were recorded.

Finally, the experimental data from these studied parameters either that recorded at the equilibrium time or over the studied time intervals were theoretically modeled to determine the suitable models describing the oil sorption process.
